# Systemic inflammation plays a key prognostic role in patients with head and neck cancer treated with immunotherapy and is linked to CT-based body composition metrics

**DOI:** 10.1007/s00405-026-10159-2

**Published:** 2026-03-15

**Authors:** Frederic Jungbauer, Sonja Ludwig, Lena Huber, Annette Affolter, Anne Lammert, Nicole Rotter, Claudia Scherl, Elena Seiz, Farroch Vahidi Noghani, Stefan O. Schönberg, Johannes Haubold, Clara Arndt, Johannes M. Ludwig

**Affiliations:** 1 Department of Otorhinolaryngology, Head and Neck Surgery, University Medical Center Mannheim, Medical Faculty Mannheim of Heidelberg University, Mannheim, 68167 Germany; 2 Department of Radiology and Nuclear Medicine, University Medical Center Mannheim, Medical Faculty Mannheim of Heidelberg University, Theodor-Kutzer-Ufer 1-3, 68167 Mannheim, Germany; 3 Department of Diagnostic and Interventional Radiology and Neuroradiology, University Hospital Essen, University of Duisburg-Essen, Essen, 45147 Germany

**Keywords:** Head and Neck Squamous Cell Cancer (HNSCC), Immunotherapy, Albumin, Body Composition Analysis (BCA), Computed Tomography (CT) Imaging, AISI (Index of Systemic Inflammation), CRP (C-Reactive Protein), CRP/L (C-Reactive Protein to Lymphocyte Ratio), dNLR (derived Neutrophil to Lymphocyte Ratio), LMR (Lymphocyte to Monocyte Ratio), NER (Neutrophil to Eosinophil Ratio), NLR (Neutrophil to Lymphocyte Ratio), NLPR (Neutrophil to Lymphocyte Platelet Ratio), PLR (Platelet to Lymphocyte Ratio), SII (Systemic Inflammatory Index), SIRI (Systemic Inflammatory Response Index)

## Abstract

**Purpose:**

To investigate the prognostic significance of pretreatment inflammatory markers and their association with computed tomography (CT)-based body composition (BC) metrics in patients with recurrent and/or metastatic head and neck squamous cell cancer (HNSCC) treated with immunotherapy.

**Methods:**

This retrospective, single-center study included 49 patients with HNSCC (20.4% female; median age 66 years). Eleven inflammatory markers, including the systemic-inflammatory-response-index (SIRI=(neutrophils×monocytes)/lymphocytes), derived neutrophil-to-lymphocyte ratio (dNLR=neutrophils/(leukocytes–neutrophils) were investigated. Pretreatment thoracic CTs yielded volumes of skeletal muscle (SM), visceral adipose tissue (VAT), and bone (B). Overall survival (OS) was assessed using Kaplan-Meier and Cox regression (HR: Hazard Ratio); group comparisons: Pearson’s and Mann-Whitney U.

**Results:**

The median OS was 17.6 months. A significantly lower HR was observed in males with positive p16-status, lower ECOG, and albumin > 3.4 g/dl. Regarding inflammation, low SIRI demonstrated the strongest HR of all inflammatory markers. In multivariate analysis, low SIRI (HR: 0.17, 95% CI: 0.05–0.57, *p* = 0.0041) and low albumin (HR: 0.26, 95%CI: 0.10–0.66, *p* = 0.0047) were identified as independent prognostic factors. Correlation analysis revealed a weak association between dNLR and neutrophil/leukocyte counts with SM/B (*r*=-0.39 and − 0.47) and (SM + VAT)/B (*r* = -0.36, -0.47) (*p* ≤ 0.02). Patients with low dNLR had higher SM/B (1.77 vs. 1.35) and (SM + VAT)/B (2.05 vs. 1.54) ratios than those with high dNLR (*p* < 0.0001).

**Conclusions:**

Pretreatment SIRI and serum albumin levels are strong, independent prognostic markers for survival in HNSCC patients receiving immunotherapy. While a link between systemic inflammation, mainly driven by neutrophils, and BC-parameters has been observed, its clinical relevance warrants further investigation.

**Supplementary Information:**

The online version contains supplementary material available at 10.1007/s00405-026-10159-2.

## Introduction

Head and neck squamous cell cancer (HNSCC) comprises a diverse set of malignancies associated with considerable patient suffering and high mortality rates. Historically, the standard of care for these diseases has involved a combination of therapies, including surgery, radiotherapy, and chemotherapy [1]. Recently, the HNSCC treatment paradigm has shifted dramatically with the introduction of immune checkpoint inhibitors, such as PD-1 pathway inhibitors, including Nivolumab and Pembrolizumab, which improve survival in patients with recurrent and metastatic HNSCC [2–5]. Despite these therapeutic breakthroughs, individual patient outcomes remain inconsistent, underscoring the urgent need to identify reliable prognostic biomarkers to guide treatment decisions and personalize patient care.

Chronic systemic inflammation is widely acknowledged as a critical contributor to cancer development and progression. The clinical significance of this link is clear, as evidence shows that systemic inflammation negatively impacts patient outcomes across various malignancies [6], including HNSCC [7], as a chronic inflammatory state can foster tumor growth, angiogenesis, and metastasis while simultaneously suppressing antitumor immunity. Several hematological markers, which are readily obtained through routine blood tests, have been shown to reflect the systemic inflammatory response. These markers have gained prominence as prognostic indicators in various cancers based on ratios and indices of peripheral blood cells, including neutrophils, lymphocytes, monocytes, eosinophils, and platelets, as well as C-reactive protein [8–13]. Moreover, systemic inflammation and body tissues, such as skeletal muscle and various fat tissues, have reciprocal influences. While higher muscle mass is associated with lower systemic inflammation in various cancers, including head and neck cancer [14, 15], cachexia is often associated with increased systemic inflammation [16–18] in cancer patients. However, research on the correlation between body composition and inflammation in patients with HNSCC treated with immunotherapy is limited.

This study had two objectives: first, to evaluate the prognostic significance of eleven pretreatment inflammatory markers for overall survival and time to progression; and second, to investigate the correlation between CT-based body composition markers and systemic inflammation in patients with metastatic or recurrent HNSCC treated with immunotherapy.

## Materials and methods

Fifty-three patients with metastatic or recurrent HNSCC underwent immunotherapy at our institution between August 2018 and May 2023. Of these, 49 patients had pretherapeutic blood laboratory values of inflammatory cells and C-reactive protein (CRP) available in their digital records. The final follow-up date for censoring was April 15, 2025. The ethics committee approved this retrospective study, and informed consent was waived (IRB approval number: 2024 − 892).

### Immunotherapy

Patients underwent a median of six immunotherapy cycles (overall range: 1–47), receiving either Nivolumab (Bristol-Myers Squibb, New York, New York, USA; 15 patients, median: 6, range: 1–36) or Pembrolizumab (MSD, Kenilworth, New Jersey, USA; 34 patients, median: 8, range: 1–47). The agents were administered according to the manufacturer’s protocol. All decisions regarding the initiation and discontinuation of immunotherapy were made by an interdisciplinary tumor board of experts. This board assessed treatment efficacy by defining tumor response and progression through a combined analysis of clinical and radiological data. Immune-related adverse events (irAEs) were recorded and graded using the Common Terminology Criteria for Adverse Events v5.0 (CTCAE).

### Systemic inflammatory markers

The following ratios and composite indices related to inflammation were determined: Index of Systemic Inflammation (AISI = (neutrophils × monocytes × platelets)/lymphocytes), C-reactive protein to lymphocyte ratio (CRP/L = CRP/lymphocyte ratio), derived neutrophil to lymphocyte ratio (dNLR = neutrophils/(white blood cells – neutrophils), neutrophil-to-eosinophil ratio (NER = neutrophils/eosinophils), lymphocyte to monocyte ratio (LMR = lymphocytes/monocytes), neutrophil to lymphocyte ratio (NLR = neutrophil/lymphocytes), neutrophil to lymphocyte-platelet ratio (NLPR = neutrophil/(lymphocyte × platelet ratio)), platelet to lymphocyte ratio (PLR = platelet/lymphocytes), systemic inflammatory index (SII = (neutrophils × platelets)/lymphocytes), and systemic inflammatory response index (SIRI = (neutrophils × monocytes)/lymphocytes). Cells were counted as the number per nanoliter (/nl). Laboratory data were obtained prior to the first immunotherapy treatment.

### Automated body composition analysis

**Fig. 1:**
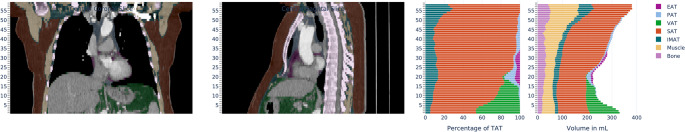
Visualization of a fully automated CT-based body composition analysis. The visual representations are furnished to illustrate the results of a fully automated body composition analysis derived from pretreatment thoracic computed tomography (CT) scans. The visualizations encompass color-coded segmentation of the distinct tissue types in both coronal and sagittal views, as well as distribution charts depicting the volume in milliliters of the total adipose tissue (TAT), visceral adipose tissue (VAT), subcutaneous adipose tissue (SAT), epicardial adipose tissue (EAT), pericardial adipose tissue (PAT), intra- and intermuscular adipose tissue (IMAT), skeletal muscle (SM), and bone, as well as the various fat fractions as a percentage of the TAT

Body composition metrics were derived from thoracic CT images using a fully automated, open-source algorithm (v0.1.3, https://github.com/UMEssen/Body-and-Organ-Analysis) as described previously [19–22]. The algorithm employs a convolutional neural network to quantify the 3D tissue volumes. Prior to analysis, all CT scans were resampled to a standard 5 mm slice thickness for uniformity. The tool calculated volumes of various tissues, including total, visceral, subcutaneous, epicardial, pericardial, and intramuscular adipose tissues (TAT, VAT, SAT, EAT, PAT, IMAT), skeletal muscle (SM), bone (B), and organs (Fig. 1). To normalize the data internally, the tissue volumes were divided by bone volume. Overall, baseline CT imaging prior to the start of immunotherapy was available for 40 patients (32 male and 8 female).

### Statistics

The median overall survival (OS) and time to progression (TTP) were estimated using the Kaplan–Meier method. The log-rank test was used to evaluate the statistical significance between groups. For inflammatory markers, receiver operating characteristic (ROC) curves with area under the curve (AUC) were calculated for 12-month survival, and the Youden index was used to determine dichotomization cutoffs (Table S1). Dichotomization was also performed by age (70 years or younger vs. older than 70 years). Both the body mass index (BMI), with cutoffs of up to 18.5 kg/m² and greater than or equal to 18.5 kg/m², and serum albumin, with cutoffs of up to 3.4 g/dL and greater than or equal to 3.4 g/dL, were set at the lower end of the normal range.

The Cox proportional hazards model was employed to estimate hazard ratios (HRs) with 95% confidence intervals (CIs) for univariate (UVA) and multivariate (MVA) risk-stratifying analyses. Factors were included in the MVA if they were statistically significant and the overall multicollinearity among all included factors was low. Variance inflation factor analysis with dummy encoding for categorical variables was performed to assess multicollinearity among the factors. Cutoffs for multicollinearity grading were set at VIF < 5 for low, VIF ≥ 5 to < 10 for moderate, and VIF ≥ 10 for high multicollinearity. Contingency analysis was conducted using Pearson’s correlation coefficients. Two groups were compared using the Mann-Whitney U-test. Owing to the exploratory nature of this study, no alpha error correction was performed. Statistical significance was set at *p* < 0.05. Statistical analyses were performed using the JMP statistical software version 18.2.2 (SAS Institute Inc., Cary, NC, USA).

## Results

### Baseline Characteristics

The present study included 49 patients (20.4% female) with a median age of 66 years (range: 40–91 years). The most prevalent tumor sites were the oropharynx (57.1%), hypopharynx (14.3%), and larynx (12.2%). Stage IV, according to the Union for International Cancer Control (UICC), was the most prevalent (61.2%), with a distant metastasis rate of 55.1%. Regarding treatment lines, immunotherapy was administered as first-line in 2.0% (n = 1), second-line in 91.8% (n = 45), and third-line in 6.1% (n = 3) of the cohort. All baseline characteristics of the study cohort are presented in Table 1.

**Table 1:** Patients’ baseline characteristics

Baseline Characteristics	Number of patients (%)
Gender • Male • Female	39 (79.6%) 10 (20.4%)
Primary tumor location • Oropharynx • Hypopharynx • Larynx • Oral cavity • Other*	28 (57.1%) 7 (14.3%) 6 (12.2%) 4 (8.2%) 4 (8.2%)
Tumor stage (UICC) • I • II • III • IV	6 (12.2%) 6 (12.2%) 7 (14.3%) 30 (61.2%)
p16-Status • Positive	15 (30.6%)
Noxious agents • None • Smoking • Alcohol • Smoking and alcohol	11 (22.4%) 16 (32.7%) 4 (8.2%) 18 (36.7%)
Previous treatments** • Surgery • Surgery + (C)RTx • CRTx • Palliative CTx/RTx • None	8 (16.3%) 20 (40.8%) 16 (32.7%) 7 (14.3%) 1 (2%)
Administered drug • Nivolumab • Pembrolizumab	15 (30.6%) 34 (69.4%)
ECOG • 0 • 1 • 2 • 3 • 4 • Unknown	16 (32.7%) 20 (40.8%) 7 (14.3%) 3 (6.1%) 2 (4.1%) 1 (2.0%)

Patients’ baseline characteristics. *The remaining tumor sites were classified as cancers of unknown primary origin, auricular cancer, nasal cancer, and parotid gland cancer. **Three patients received two prior treatments. Abbreviations: ECOG (Eastern Cooperative Oncology Group), UICC (Union for International Cancer Control), (C)RTx (Chemo-) Radiotherapy); RTx (Radiotherapy) CTx (Chemotherapy)

### Survival analysis

**Fig. 2:**
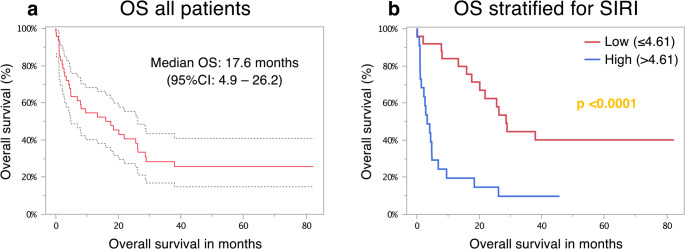
Overall survival of the study cohort and stratified for SIRI. Overall survival of the study cohort (**a**) with 95% confidence interval (95% CI; dotted line) and according to high (> 4.61) and low (≤ 4.61) Systemic Inflammatory Response Index (SIRI) (**b**) following the first treatment with immunotherapy. A log-rank test was used for the statistical analysis

In thirty-three patients, death was documented after a median interval of 4.9 months (range: 0.13–37.97 months), while sixteen patients were censored at the end of the follow-up period or were lost to follow-up after a median of 42.4 months (range: 0–82.3 months). The estimated median overall survival in this study for all patients was 17.6 months (95% CI: 4.9–26.2), with 1-month, 3-month, 6-month, 1-year, 2-year, and 3-year survival rates of 91.8%, 71.4%, 61.2%, 51.0%, 36.7%, and 24.5%, respectively (Fig. 2a).

To dichotomize inflammatory markers, AUC analysis of 12-month survival was performed to determine the cutoffs (Table S1). Except for NER, the calculated area under the curve (AUC) was statistically significant, with an AUC of up to 0.875 (95% CI: 0.749–0.952, *p* < 0.0001) for SIRI. Table 2 presents the median overall survival rates for the clinical and inflammatory markers, along with the results of the Cox proportional hazards uni- and multivariate analysis. Among all inflammatory markers, only NER was not statistically significant. Low SIRI (≤ 4.61) emerged as the strongest factor, exhibiting the lowest hazard ratio of 0.241 (0.116–0.499) and a prolonged median OS of 28.6 months. In contrast, patients with high SIRI exhibited a median survival of only 3.4 months (*p* = 0.0001) (Fig. 2b). Among the included clinical factors, sex, p16-status, ECOG stage, and serum albumin were statistically significant (Table 2).

**Table 2:** Univariate and multivariate survival analysis of clinical parameters and inflammatory markers

Overall survival analysis			Univariate analysis		Multivariate analysis	
**Groups**		**Median overall survival in months (95% CI)**	**HR (95% CI)**	***p*** **-value**	**HR (95% CI)**	***p*** **-value**
Sex	Female	4.8 (0.87–21.9)	1	**0.026**	1	0.79
	Male	20.2 (7.93–38.0)	0.38 (0.17–0.84)		0.87 (0.31–2.43)	
Age	> 70 years	20.2 (4.2 – .)	1	0.24	-	-
	≦ 70 years	13.4 (2.1–26.3)	0.66 (0.33–1.31)		-	
p16-status	Negative	13.4 (3.4–21.9)	1	**0.029**	1	0.96
	Positive	Not reached (6.9 - .)	0.42 (0.18–0.97)		1.02 (0.39–2.69)	
UICC	1	Not reached (0.23 – .)	1	0.81	-	-
	2	18.5 (1.1–38.0)	1.83 (0.44–7.7)		-	
	3	21.6 (0.13 – .)	1.4 (0.31–6.24)		-	
	4	13.4 (4.2–26.3)	1.66 (0.49–5.57)		-	
ECOG	0	37.97 (1.2 – .)	1	**0.018**	1	0.56
	1	17.6 (4.2–28.6)	1.9 (0.79–4.6)		1.34 (0.54–3.33)	
	2	6.93 (0.23–21.9)	4.7 (1.5–14.6)		2.35 (0.67–8.23)	
	3–4	1.5 (1.1 - .)	6.13 (1.67–22.5)		2.23 (0.49–10.09)	
BMI	< 18.5 kg/m^2)^	3.9 (1.1–20.2)	1	0.15	-	-
	≥ 18.5 kg/m^2)^	18.5 (6.93–28.63)	0.51 (0.22–1.22)		-	
Albumin	≦ 3.4 g/dl	4.5 (2.1–21.9)	1	**0.0029**	1	**0.0047**
	> 3.4 g/dl	37.97 (16 – .)	0.34 (0.16–0.71)		0.26 (0.10–0.66)	
NLR	High (> 6.59)	4.2 (1.5–7.93)	1	**0.0005**	-	-
	Low (≤ 6.59)	28.9 (17.6 – .)	0.283 (0.136–0.586)		-	
AISI	High (> 1618.4)	3.4 (1.13–4.83)	1	**0.0007**	-	
	Low (≤ 1618.4)	26.3 (17.6 – .)	0.277 (0.135–0.568)		-	
LMR	High (> 0.978)	26.3 (16 – .)	0.276 (0.132–0.573)	**0.0009**	-	-
	Low (≤ 0.978)	4.2 (2.1–4.93)	1		-	
SIRI	High (> 4.61)	3.4 (1.2–4.93)	1	**0.0001**	1	**0.0041**
	Low (≤ 4.61)	28.6 (17.633 – .)	0.241 (0.116–0.499)		0.17 (0.05–0.57)	
CRP	High (> 15 mg/L)	4.2 (2.1–17.6)	1	**0.007**	-	-
	Low (≤ 15 mg/L)	26.2 (8.1 – .)	0.36 (0.18–0.76)		-	
CRP/L	High (> 17.49)	4.83 (2.33–21.9)	1	**0.0148**	-	-
	Low (≤ 17.49)	32.083 (16.0 - .)	0.389 (0.176–0.86)		-	
dNLR	High (> 3.93)	4.5 (1.1–7.93)	1	**0.0161**	-	-
	Low (≤ 3.93)	21.9 (13.37 – .)	0.41 (0.204–0.827)		-	
NLPR	High (> 0.029)	4.5 (1.2–7.93)	1	**0.0007**	1	0.84
	Low (≤ 0.029)	28.63 (16 – .)	0.283 (0.138–0.579)		0.9 (0.31–2.59)	
PLR	High (> 318.5)	4.83 (2.33–9.6)	1	**0.0185**	-	-
	Low (≤ 318.5)	28.63 (16 – .)	0.429 (0.209–0.882)		-	
SII	High (> 147.97)	4.2 (1.5–6.93)	1	**0.0017**	-	-
	Low (≤ 147.97)	28.63 (17.63 – .)	0.32 (0.153–0.653)		-	
NER	High (> 638.6)	18.47 (2.7–26.33)	1	0.667	-	-
	Low (≤ 638.6)	17.63 (7.93–28.93)	1.167 (0.579–2.352)		-	

Univariate and Multivariate Survival Analyses of Clinical Parameters and Inflammatory Markers. Abbreviations: AISI (Aggregate Index of Systemic Inflammation), BMI (Body mass index), CRP (C-Reactive Protein), CRP/L (C-Reactive Protein/Lymphocyte ratio), dNLR (derived Neutrophil-to-Lymphocyte Ratio), ECOG (Eastern Cooperative Oncology Group), LMR (Lymphocyte-to-Monocyte Ratio), NLPR (Neutrophil-to-Lymphocyte-to-Platelet Ratio), NLR (Neutrophil-to-Lymphocyte Ratio), SIRI (Systemic Inflammatory Response Index), UICC (Union for International Cancer Control)

Given the substantial overlap among inflammatory markers, a variance inflation factor analysis was performed, revealing moderate to high multicollinearity among all inflammatory factors except NLPR. Among the factors exhibiting moderate to high collinearity, SIRI was selected for the multivariate analysis, as it demonstrated the lowest hazard ratio and the lowest p-value. In the VIF analysis, only low multicollinearity (VIF < 5) was observed among the factors included in the multivariate hazard ratio analysis. Here, only low SIRI (HR: 0.17, 95% CI: 0.05–0.57; *p* = 0.0041) and serum albumin levels > 3.4 g/dl (HR: 0.26, 95% CI: 0.10–0.66; *p* = 0.0047) were confirmed as significant independent prognostic factors (Table 2).

### Evaluation of the relationship between inflammatory markers and CT-based body composition measurements

**Fig. 3:**
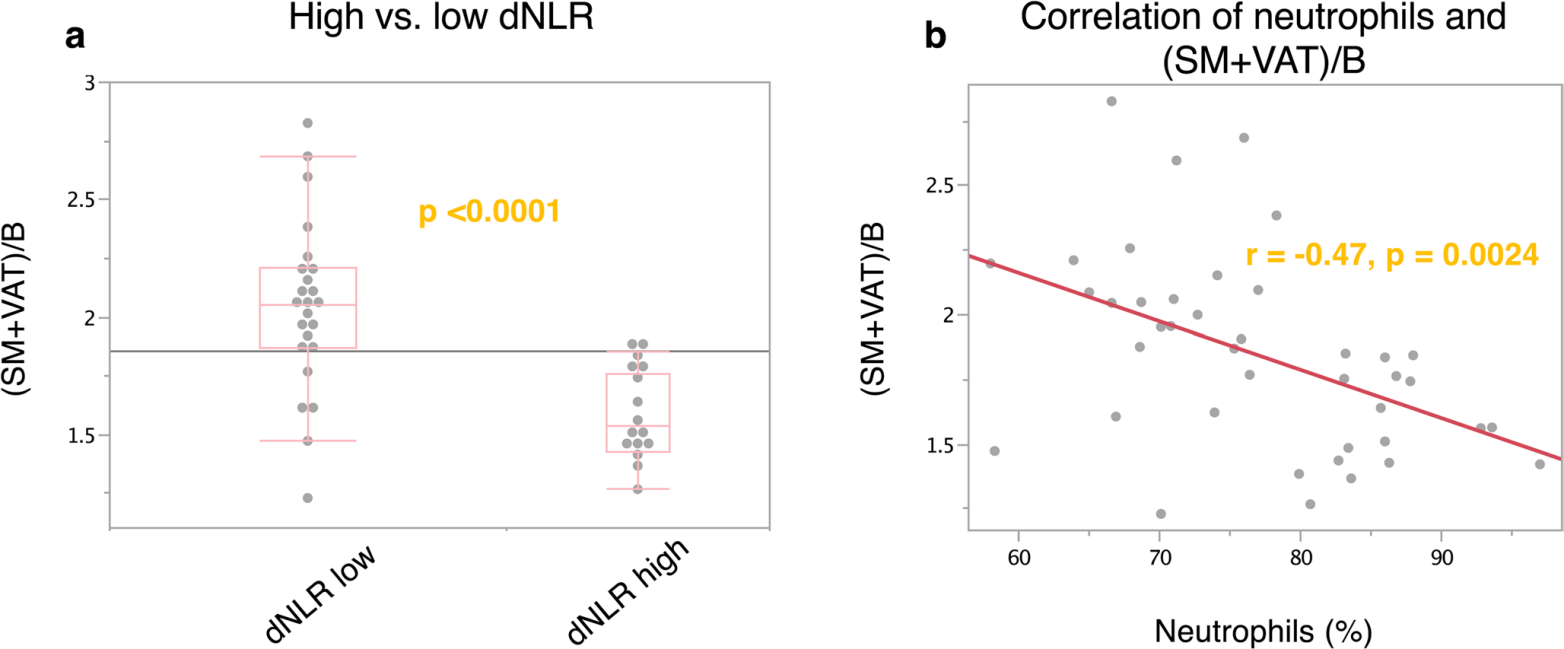
Association of (SM + VAT)/B with dNLR and neutrophils. The association of (SM + VAT)/B with dNLR and neutrophils was examined. Significantly higher (SM + VAT)/B values were observed for patients with low (≤ 3.93) dNLR (2.05, 95% CI: 1.88–2.20) than for patients with high (> 3.93) dNLR (1.54, 95% CI: 1.43–1.75) (*p* < 0.0001) (Figure a). The neutrophil fraction of all leukocytes significantly correlates with (SM + VAT)/B (*r* = -0.47, *p* = 0.0024) (Figure b)

In an exploratory analysis of the relationship between inflammatory markers and CT-based body composition markers, significantly higher SM/B and (SM + VAT)/B ratios were observed in patients with low NLR, dNLR, AISI, and SII (Table 3; Fig. 3a). Consistent with established physiological norms, males presented with significantly elevated ratios for (SM + VAT)/B (m: 1.94 (95% CI: 1.8–2.08), f: 1.48 (95% CI: 1.36–1.61), *p* = 0.001) and SM/B (m: 1.68 (95% CI: 1.57–1.78), f: 1.34 (95% CI: 1.16–1.52), *p* = 0.005) relative to females. Upon stratification by sex, only the (SM + VAT)/B ratio remained statistically significantly associated with both dNLR and SII in the male and female cohorts. An analysis of continuous variables identified a weak inverse correlation between the inflammatory indices dNLR and NLPR and the body composition parameters SM/B and (SM + VAT)/B. However, when stratified by sex, this statistical significance was observed only for the relationship between dNLR and the (SM + VAT)/B ratio in the male subgroup (Table S2).

**Table 3:** Comparison of high and low inflammation and albumin levels in relation to muscle-to-bone (SM/B) and muscle-plus-visceral adipose tissue-to-bone (SM+VAT/B) ratios

Parameters		SM/B					
		All		Male		Female	
		Median (IQR)	*p*-value	Median (IQR)	*p*-value	Median (IQR)	*p*-value
SIRI	High	1.51 (1.33–1.75)	0.15	1.69 (1.49–1.82)	0.57	1.32 (1.16–1.35)	0.096
	Low	1.70 (1.48 – 1.93)		1.71 (1.61–1.93)		1.58 (1.51–1.64)	
Albumin	High	1.69 (1.48 – 1.93)	0.16	1.70 (1.62–1.94)	0.25	1.23 (1.13–1.34)	0.51
	Low	1.51 (1.32 - 1.75)		1.68 (1.44–1.81)		1.34 (1.31–1.43)	
NLR	High	1.5 (1.32 – 1.71)	0.017	1.60 (1.44–1.71)	0.08	1.33 (1.21–1.40)	0.5
	Low	1.79 (1.57 – 1.95)		1.80 (1.67–1.94)		1.60 (1.44–1.71)	
dNLR	High	1.35 (1.30–1.50)	<0.0001	1.49 (1.32–1.63)	0.002	1.32 (1.16–1.35)	0.096
	Low	1.77 (1.67–1.94)		1.80 (1.67–1.94)		1.58 (1.51–1.64)	
AISI	High	1.48 (1.32–1.70)	0.041	1.68 (1.48–1.73)	0.33	1.32 (1.16–1.35)	0.096
	Low	1.71 (1.53–1.92)		1.74 (1.59–1.93)		1.58 (1.51–1.64)	
SII	High	1.48 (1.32–1.68)	0.008	1.53 (1.41–1.72)	0.067	1.32 (1.16–1.35)	0.096
	Low	1.74 (1.66–1.93)		1.79 (1.67–1.94)		1.58 (1.51–1.64)	
		SM + VAT/B					
		All		Male		Female	
		Median (IQR)	*p*-value	Median (IQR)	*p*-value	Median (IQR)	*p*-value
SIRI	High	1.75 (1.45–1.94)	0.041	1.86 (1.70–2.01)	0.19	1.43 (1.38–1.47)	0.046
	Low	2.00 (1.67–2.19)		2.05 (1.79–2.20)		1.69 (1.65–1.73)	
Albumin	High	1.95 (1.84–2.17)	0.031	1.81 (1.56–2.09)	0.11	1.52 (1.47–1.56)	0.74
	Low	1.69 (1.45–1.96)		1.81 (1.56–2.09)		1.46 (1.39–1.50)	
NLR	High	1.74 (1.46–1.86)	0.003	1.84 (1.62–1.92)	0.015	1.61 (1.61–1.61)	0.51
	Low	2.06 (1.88–2.21)		2.07 (1.94-2.22)		1.61 (1.61–1.61)	
dNLR	High	1.54 (1.43–1.75)	<0.0001	1.69 (1.56–1.82)	<0.0001	1.43 (1.38–1.47)	0.046
	Low	2.05 (1.88–2.20)		1.54 (1.43–1.75)		1.69 (1.65–1.73)	
AISI	High	1.57 (1.43–1.88)	0.007	1.84 (1.57–1.95)	0.07	1.43 (1.38–1.47)	0.046
	Low	2.00 (1.77–2.20)		2.05 (1.86–2.20)		1.69 (1.65–1.73)	
SII	High	1.64 (1.44-1.85)	0.006	1.84 (1.60–1.98)	0.03	1.69 (1.65–1.73)	0.046
	Low	2.05 (1.82–2.20)		2.06 (1.88–2.21)		1.69 (1.65–1.73)	

Table 3 Comparison of high and low inflammation and albumin levels in relation to the muscle-to-bone (SM/B) and muscle-plus-visceral adipose tissue-to-bone (SM+VAT/B) ratios. No statistical significance was observed for LMR, CRP/L, CRP, NER, or PLR

To better understand the effects of immune cell subsets, an exploratory analysis of peripheral immune cells was performed, revealing a correlation between SM/B and leukocytes/nl (-0.41, *p* = 0.009) as well as neutrophils/nl (-0.42, *p* = 0.007), but not with lymphocytes (0.09, *p* = 0.58) or other immune cell subsets. A similar significant correlation between (SM + VAT)/B was also seen for leukocytes/nl (-0.32, *p* = 0.04) and neutrophils/nl (-0.35, *p* = 0.028), but not for lymphocytes/nl (0.26, *p* = 0.1). Interestingly, the correlation coefficients were higher for the relative cell proportions of all leukocytes in percent, with − 0.47 (*p* = 0.002) for neutrophils, 0.4 (*p* = 0.016) for lymphocytes, and 0.5 (*p* = 0.001) for monocytes when correlated with SM/B. Similar findings were observed for SM + VAT/B regarding neutrophils (-0.47, *p* = 0.0024, Fig. 3b), lymphocytes (0.44, *p* = 0.004), and monocytes (0.43, *p* = 0.006). It should be noted that a statistical correlation was observed only in men, but not in women, for neutrophils per nanoliter and percentages for SM/B and SM + VAT/B (correlation coefficient range: -0.37 to -0.46); Supplementary Table S3 provides further details. Of note, for VAT/B, only a weak correlation was observed for lymphocytes/nl (*r* = 0.35, *p* = 0.026), with significance in males (*r* = 0.4, *p* = 0.024) but not in females.

#### Time to progression analysis

The median time to progression was 11.6 months (95% CI: 3.17–14.5) with 1-, 3-, and 6-month and 1-, 2-, and 3-year progression-free rates of 88.4%, 65.1%, 58.1%, 39.5%, 25.6%, and 18.6%, respectively. The time to progression analysis regarding inflammatory markers and clinical parameters is shown in Table S4, where high LMR had the lowest hazard ratio of 0.237 (95% CI: 0.103–0.547, *p* = 0.0012), followed by low SIRI with a hazard ratio of 0.261 (95% CI: 0.118–0.57, *p* = 0.001). In the multivariate risk factor analysis, only the inflammatory markers LMR, CRP, and NLPR were included, while those with moderate or high multicollinearity were excluded. In particular, SIRI and LMR showed high collinearity (*r* = -0.73, *p* < 0.001); therefore, SIRI was omitted from the TTP. In the MVA, only low LMR (0.173, 95% CI: 0.041–0.73, *p* = 0.017) and ECOG 0 performance status (ECOG 0: 1, ECOG 1: 1.44, 95% CI: 0.51–4.11; ECOG 2: 1.68, 95% CI: 0.37–7.69, ECOG 3: 12.4, 95% CI: 2.07–74.44; *p* = 0.043) remained statistically significant (Table S4).

#### Immune-related adverse events

Immune-related adverse events (irAEs) were reported in 30.6% of the patient cohort (*n* = 15). These side effects first appeared a median of 4 weeks after treatment commencement (range: 2–60 weeks). Except for a single case of grade 3 autoimmune vasculitis and hepatitis, all other irAEs were mild (grade 1–2). Furthermore, the analysis demonstrated that the occurrence of these adverse events was independent of the inflammation markers studied.

## Discussion

The present study unequivocally demonstrated the general prognostic significance of inflammatory markers for overall survival and time to progression in patients with recurrent and/or metastatic HNSCC treated with immunotherapy. Among the evaluated hematologic inflammatory markers, SIRI was identified as the most significant risk factor for survival, with prolonged survival associated with lower SIRI values. SIRI was initially proposed by Qi et al. to predict survival in patients with pancreatic cancer who underwent chemotherapy. It is a measure of inflammation based on neutrophil, monocyte, and lymphocyte levels in the blood, which comprehensively reflects the host’s pro- and anticancer immune balance [23]. Subsequent studies have demonstrated the predictive usefulness of SIRI in various cancers [24], including HNSCC [25–27]. The rationale underlying this approach is that elevated levels of peripheral neutrophils and monocytes are associated with poor clinical outcomes in patients diagnosed with head and neck squamous cell carcinoma (HNSCC) and other forms of cancer [28]. Conversely, lymphopenia, indicative of a compromised immune system’s capacity to impede tumor proliferation, is associated with a more unfavorable prognosis [29, 30]. Overall, the SIRI index serves as a quantitative indicator of the equilibrium between pro-tumorigenic and anti-tumorigenic immune cell populations, with lower SIRI values correlating with improved clinical prognosis. The determined cutoff for SIRI with ≤ 4.61 in this study was evidently higher than those observed in previous studies on patients with head and neck cancer treated with either or a combination of surgery, radiotherapy, and chemotherapy, ranging from 0.84 in nasopharyngeal to 3.26 in patients with laryngeal cancer, both treated with and without chemotherapy [25, 26, 31–33]. Overall, SIRI values in this cohort were notably higher than previously reported, with a median SIRI of 4.4. Specifically, the lowest observed value in our study was 1.19, whereas approximately 30% of patients in Valero et al. had values below 1.10 [26]. Furthermore, 68.8% of our cohort had SIRI values > 2.8, compared to only ~ 20% in the reference study. Furthermore, our study revealed a median overall survival (OS) of 17.6 months and an estimated 5-year survival rate of 6.1%, both of which signify a substantially poorer prognostic outcome, as these findings stand in stark contrast to previous reports, such as those by Atasever Akkas et al., who documented a median OS of 47 months for patients with SIRI values exceeding 1.58 [25]. Similarly, our results diverge from the 5-year disease-specific survival rates of 47.4% reported by Valero et al. (SIRI > 2.8) [26] and 27.6% by Chuang et al. (SIRI > 3.26) [32]. The observed discrepancy may be explained by the distinct clinical characteristics of the study cohort, which was predominantly composed of patients with advanced-stage and recurrent disease and a high inflammatory status who had failed prior curative treatment. This contrasts with the primarily curative-intent populations assessed in previous studies, potentially also accounting for the elevated SIRI values in our cohort. Therefore, it can be hypothesized that these thresholds may also have limited applicability in first-line immunotherapy settings, such as the Keynote-689 cohort, given the significant clinical disparities of the study populations. This emphasizes the need for additional research into the relevance and optimal prognostic cutoffs of inflammatory markers for patients undergoing immunotherapy as part of first-line treatment [34]. Nevertheless, the present data suggest that, even in patients receiving immunotherapy as palliative treatment, the SIRI index remains a clinically relevant parameter for stratifying individuals by prognosis and distinguishing those with favorable versus poor survival and time to progression.

While SIRI emerged as the most significant prognostic discriminator for overall survival, other inflammatory markers, notably NLR and LMR, demonstrated comparable, albeit slightly weaker, prognostic value. Nevertheless, a definitive conclusion regarding the superiority of one marker over the other cannot be drawn from these results based on the study cohort. However, a potential explanation for SIRI’s enhanced performance is its composite nature, which integrates NLR and LMR into a single metric. Furthermore, each of the immune cell populations incorporated into the SIRI index, namely neutrophils, lymphocytes, and monocytes, was independently identified as a significant predictor of overall survival in the receiver operating characteristic analysis (Table S1).

Regarding time-to-progression, the majority of inflammatory markers were significant in univariate analysis. High LMR emerged as the strongest predictor of prolonged time to progression, followed by low SIRI. This finding aligns with previous research, including a meta-analysis [35, 36], and underscores the pivotal role of host immune competence. LMR serves as a surrogate for this competence and is significantly positively associated with intratumoral infiltration of CD4 + and CD20 + lymphocytes, but not with tumor-infiltrating monocytes [37].

In line with previous research on HNSCC patients receiving immunotherapy, this study confirmed that hypoalbuminemia is a predictor of both overall survival and time to progression [12, 13, 38]. The prognostic significance of low albumin levels is likely tied to its role as a marker of systemic inflammation, metabolic dysregulation, and overall disease severity [39]. Furthermore, a direct pharmacokinetic relationship has been established. Pembrolizumab and Nivolumab metabolism is intrinsically linked to the neonatal Fc receptor pathway, which is also responsible for maintaining serum albumin levels. Therefore, low albumin levels may indicate increased protein catabolism, accelerating the breakdown of these drugs and resulting in lower therapeutic concentrations [40]. This reduced drug exposure can lead to diminished treatment efficacy and, consequently, shorter survival times, as observed in this cohort.

Analysis of the associations among inflammatory markers, immune cell populations, and body composition parameters demonstrated that patients with low dNLR values exhibited significantly higher (SM + VAT)/B and SM/B ratios. Furthermore, the strongest yet still weak negative correlation was observed between the continuous variables dNLR and relative neutrophil counts with (SM + VAT)/B and SM/B. Given the exponential correlation between relative neutrophil counts and dNLR, this underscores the predominant association between neutrophils and body composition parameters. This aligns with the established role of neutrophils as a hallmark of tumor-associated cachexia, wherein they contribute to a pro-inflammatory microenvironment characterized by elevated levels of cytokines, such as interleukin-6 (IL-6), interleukin-8 (IL-8), and tumor necrosis factor-alpha (TNF-α), which drive catabolic processes in skeletal muscle and adipose tissue [16–18]. Consistently, patients with cachexia exhibit increased serum concentrations of neutrophil activation markers (e.g., calprotectin, myeloperoxidase, and elastase) and neutrophil-secreted proteins such as lipocalin-2, which are associated with poor nutritional status and the degree of systemic inflammation [17, 41]. Furthermore, myocytes and adipocytes exert immune regulatory functions by secreting a broad spectrum of cytokines, including myokines and adipokines, which can either enhance or attenuate inflammatory signaling cascades [42, 43]. This bidirectional interaction underscores the reciprocal influence between the immune system and both the skeletal muscle and adipose tissue compartments.

A recent meta-analysis demonstrated a correlation between increased VAT and enhanced overall survival and progression-free survival in patients with various cancers undergoing treatment with immune checkpoint inhibitors, highlighting its prognostic relevance [44]. This study identified a weak positive correlation between the visceral adipose tissue to bone ratio (VAT/B) and peripheral lymphocyte counts. Notably, stratification of patients according to high versus low levels of inflammatory ratios and indices, specifically those contrasting neutrophil and lymphocyte populations, revealed greater statistical significance for (SM + VAT)/B than for SM/B. These findings suggest that including VAT in the ratio may amplify the association with inflammatory status, despite including only visceral adipose tissue from the upper abdominal region. Of note, continuous VAT/B alone was not a statistically significant OS factor in the Cox proportional hazard model, whereas continuous (SM + VAT)/B (HR: 0.288, 95% CI: 0.11–0.66, *p* = 0.005) and continuous SM/B (HR: 0.188, 95% CI: 0.053–0.67, *p* = 0.009) ratios were.

The weak-to-modest correlations identified between body composition and inflammatory markers in the present study suggest a complex, multifactorial etiology. We hypothesize that other variables, including immune cell phenotypes, diverse pro- and anti-inflammatory mediators, genetic predispositions, and physical activity, are significant co-determinants in the regulation of skeletal muscle and visceral adipose tissue volumes, as well as systemic inflammation. Future investigations should aim to delineate the causal pathways underlying this observation and ascertain its physiological and clinical significance.

Several limitations warrant consideration. The retrospective, monocentric nature of this study, coupled with the limited cohort size, restricts the external validity of our conclusions. This is particularly salient regarding the skewed sex distribution, as the low number of female participants rendered any sex-stratified analysis statistically underpowered, thereby precluding the ability to either robustly confirm or exclude sex-specific prognostic significance, especially in women. Consequently, external validation of the findings is warranted. It is important to consider the possibility of another selection bias, which may be present in the selection process of the inflammatory markers included in the multivariate analysis. This consideration is particularly relevant in light of the relatively small study cohort and the absence of a clear significance of SIRI over the others. Furthermore, the incomplete availability of pretreatment computed tomography (CT) scans for all patients constituted a potential source of selection bias. This could specifically affect the analysis of associations between immune cell populations and body composition parameters, underscoring the need to validate these findings in a larger, ideally prospective cohort.

## Conclusion

This study concluded that pretreatment inflammatory markers have prognostic value in HNSCC patients receiving immunotherapy for metastatic or recurrent disease. In this patient population, the Systemic Inflammation Response Index (SIRI) and serum albumin levels have been identified as robust, independent prognostic factors for survival. Although not yet considered the standard of care, mounting evidence supports the use of these markers to enhance risk stratification, facilitating the identification of high-risk patients who may require more intensive surveillance or alternative treatment strategies. While a statistically significant association between inflammation and body composition was identified, future investigations should aim to delineate the causal pathways underlying this observation and ascertain its physiological and clinical significance.

Table 3: Comparison of high and low inflammation and albumin levels in relation to the muscle-to-bone (SM/B) and muscle-plus-visceral adipose tissue-to-bone (SM + VAT/B) ratios. No statistical significance was observed for LMR, CRP/L, CRP, NER, or PLR.

## Supplementary Information

Below is the link to the electronic supplementary material.

Supplementary Material 1

## Abbreviations

AISI: Index of Systemic Inflammation
AUC: Area Under the Curve
B: Bone
BCA: Body Composition Analysis
BMI: BMI
CI: Confidence Intervals
CRP: C Reactive Protein
CRP/L: C Reactive Protein to Lymphocyte ratio
(C)RTx: (Chemo) Radiotherapy
CT: Computed Tomography
CTCAE: Common Terminology Criteria for Adverse Events
CTx: Chemotherapy
dNLR: derived Neutrophil to Lymphocyte Ratio
EAT: Epicardial Adipose Tissue
ECOG: Eastern Cooperative Oncology Group
HNSCC: Head and Neck Squamous Cell Cancer
HR: Hazard Ratio
IMAT: Intramuscular Adipose Tissue
IQR: Interquartile Ranges
irAEs: Immune related Adverse Events
LMR: Lymphocyte to Monocyte Ratio
MVA: Multivariate Analysis
NER: Neutrophil to Eosinophil Ratio
NLPR: Neutrophil to Lymphocyte Platelet Ratio
NLR: Neutrophil to lymphocyte ratio
OS: Overall Survival
PAT: Pericardial Adipose Tissue
PD-1: Programmed Death-1
PLR: Platelet to Lymphocyte Ratio
ROC: Receiver Operating Curve
RTx: Radiotherapy
SAT: Subcutaneous Adipose Tissue
SII: Systemic Inflammatory Index
SIRI: Systemic Inflammatory Response Index
SM: Skeletal Muscle
TAT: Total Adipose Tissue
TTP: Time to Progression
UICC: Union for International Cancer Control
UVA: Univariate Analysis
VAT: Visceral Adipose Tissue
VIF: Variance Inflation Factor
