# Supplementary material for: Systemic inflammation plays a key prognostic role in patients with head and neck cancer treated with immunotherapy and is linked to CT-based body composition metrics
Source: Eur Arch Otorhinolaryngol. 2026 Mar 15;283(5):3361–71. doi: 10.1007/s00405-026-10159-2 (PMC13152974; doi:10.1007/s00405-026-10159-2)
Supplement: Supplementary file 1 — Supplementary Material 1 [file 405_2026_10159_MOESM1_ESM.docx]

**Supplementary materials:**

**Table S1:** Receiver Operating Curve (ROC) analysis of inflammatory markers and immune cells

| **Parameter** | **AUC (95%CI)** | **Cutoff 12 months** | **Sensitivity/Specificity (%)** | **Youdens Index** | **p-value** |
| --- | --- | --- | --- | --- | --- |
| NLR | 0.855 (0.725 – 0.939) | ≤6.586776859 | 79.17/80.0 | 0.5917 | <0.0001 |
| AISI | 0.838 (0.705 – 0.928) | ≤1618.42736 | 87.5 / 72.0 | 0.595 | <0.0001 |
| CRP | 0.661 (0.516 – 0.786) | ≤15 | 62.5 / 75.0 | 0.378 | 0.036 |
| CRP/L | 0.741 (0.589 – 0.86) | ≤17.487499676 | 63.64 / 86.96 | 0.5059 | 0.0014 |
| dNLR | 0.814 (0.677 – 0.911) | ≤3.926108374 | 87.5 / 60.0 | 0.4750 | <0.0001 |
| LMR | 0.787 (0.646 – 0.891) | >0.977941177 | 91.67 / 68.00 | 0.5967 | <0.0001 |
| NER | 0.569 (0.419 – 0.711) | ≤638.55554332 | 66.67 / 58.33 | 0.25 | 0.41 |
| NLPR | 0.752 (0.608 – 0.864) | ≤0.028952703 | 83.33 / 64.00 | 0.4733 | 0.0004 |
| PLR | 0.778 (0.637 – 0.884) | ≤318.54379977 | 70.83 / 84.00 | 0.8483 | <0.0001 |
| SII | 0.837 (0.703 – 0.927) | ≤147.96790244 | 79.17 / 80.00 | 0.5917 | <0.0001 |
| SIRI | 0.875 (0.749 – 0.952) | ≤4.609769268 | 87.5 / 80.00 | 0.6750 | <0.0001 |
| Neutrophils % | 0.814 (0.677 – 0.911) | ≤79.7 | 87.5 / 60.0 | 0.4750 | <0.0001 |
| Lymphocytes % | 0.858 (0.728 – 0.941) | ≤9.2 | 87.5 / 72.00 | 0.595 | <0.0001 |
| Monocytes % | 0.642 (0.492 – 0.774) | ≤5.2 | 100 / 28.00 | 0.2800 | 0.074 |
| Eosinophils % | 0.641 (0.491 – 0.733) | ≤2.0 | 58.33 / 68.00 | 0.2633 | 0.0757 |
| Basophils % | 0.724 (0.578 – 0.842) | ≤0.3 | 87.5 / 52.00 | 0.3950 | 0.0024 |
| Leukocytes /nl | 0.718 (0.571 – 0.837) | ≤7.0 | 70.83 / 68.0 | 0.3883 | 0.0028 |
| Neutrophils /nl | 0.76 (0.617 – 0.870) | ≤4.64212 | 62.05 / 84.00 | 0.4650 | 0.0001 |
| Lymphocytes /nl | 0.718 (0.572 – 0.837) | ≤1.04598 | 50.00 / 92.00 | 0.4200 | 0.0029 |
| Monocytes /nl | 0.570 (0.421 – 0.711) | ≤0.73743 | 83.33 / 40.0 | 0.2333 | 0.3988 |
| Eosinophils / nl | 0.55 (0.41 – 0.692) | ≤0.161 | 50.00 / 72.00 | 0.2200 | 0.5498 |
| Basophil / nl | 0.628 (0.478 – 0.761) | ≤0.0312 | 66.67 / 64.00 | 0.3067 | 0.1134 |
| Thrombocytes /nl | 0.671 (0.528 – 0.794) | ≤216 | 44.00 / 89.29 | 0.3329 | 0.0228 |
| SIRI / Albumin | 0.888 (0.760 – 0.960) | >0.121309718 | 83.33 / 88.00 | 0.7133 | <0.0001 |

Receiver Operating Curve (ROC) analysis of inflammatory markers and immune cells, with results from cutoff selection set at a 12-month survival. Abbreviations: AUC, Area Under the Curve.

**Table S2:** Correlation analysis of inflammatory markers and albumin levels with muscle-to-bone (SM/B) and muscle-plus-visceral adipose tissue-to-bone (SM+VAT/B) ratios.

| Parameters | **SM/B** | | | | | |
| --- | --- | --- | --- | --- | --- | --- |
|  | **All** | | **Male** | | **Female** | |
|  | Correlation | p-value | Correlation | p-value | Correlation | p-value |
| AISI | -0.15 | 0.35 | -0.141 | 0.45 | -0.517 | 0.16 |
| CRP | -0.302 | 0.06 | -0.404 | 0.024 | 0.167 | 0.67 |
| CRP/L | -0.159 | 0.33 | -0.245 | 0.18 | 0.37 | 0.33 |
| LMR | 0.038 | 0.82 | -0.111 | 0.55 | 0.386 | 0.3 |
| NER | -0.239 | 0.14 | -0.343 | 0.06 | -0.412 | 0.27 |
| NLPR | -0.324 | **0.039** | -0.405 | 0.02 | 0.134 | 0.73 |
| NLR | -0.283 | 0.073 | -0.326 | 0.07 | -0.342 | 0.37 |
| PLR | -0.164 | 0.31 | -0.157 | 0.4 | -0.489 | 0.18 |
| SII | -0.214 | 0.18 | -0.262 | 0.15 | -0.352 | 0.35 |
| SIRI | -0.209 | 0.19 | -0.206 | 0.26 | -0.538 | 0.14 |
| dNLR | -0.39 | **0.012** | -0.449 | 0.01 | -0.35 | 0.36 |
| Albumin | 0.31 | 0.06 | 0.38 | **0.031** | -0.34 | 0.42 |
|  |  |  |  |  |  |  |
|  | **(SM+VAT)/B** | | | | | |
|  | **All** | | **Male** | | **Female** | |
|  | Correlation | p-value | Correlation | p-value | Correlation | p-value |
| AISI | -0.228 | 0.15 | -0.235 | 0.1947 | -0.644 | 0.061 |
| CRP | -0.252 | 0.12 | -0.326 | 0.0732 | 0.255 | 0.508 |
| CRP/L | -0.214 | 0.18 | -0.313 | 0.0865 | 0.593 | 0.0921 |
| LMR | 0.15 | 0.36 | 0.047 | 0.7992 | 0.253 | 0.5111 |
| NER | -0.253 | 0.12 | -0.362 | **0.045** | -0.383 | 0.3089 |
| NLPR | -0.343 | **0.0279** | -0.442 | 0.011 | 0.506 | 0.1642 |
| NLR | -0.337 | 0.03 | -0.401 | 0.023 | -0.028 | 0.9434 |
| PLR | -0.26 | 0.1 | -0.283 | 0.12 | -0.304 | 0.4256 |
| SII | -0.261 | 0.1 | -0.319 | 0.075 | -0.43 | 0.2483 |
| SIRI | -0.28 | 0.076 | -0.298 | 0.097 | -0.525 | 0.1464 |
| dNLR | -0.358 | **0.0217** | -0.416 | **0.018** | -0.119 | 0.7607 |
| Albumin | 0.38 | 0.017 | 0.41 | **0.02** | -0.15 | 0.73 |

**Table S3:** Correlation analysis of inflammatory cells and platelets with muscle-to-bone (SM/B) and muscle-plus-visceral adipose tissue-to-bone (SM+VAT/B) ratios.

| Parameters | **SM/B** | | | | | |
| --- | --- | --- | --- | --- | --- | --- |
|  | **All** | | **Male** | | **Female** | |
|  | Correlation | p-value | Correlation | p-value | Correlation | p-value |
| Leukocytes/nl | -0.41 | **0.009** | -0.46 | **0.0087** | -0.29 | 0.49 |
| Neutrophils/nl | -0.42 | **0.007** | -0.46 | **0.0086** | -0.31 | 0.46 |
| Lymphocytes/nl | 0.09 | 0.58 | 0.04 | 0.84 | 0.06 | 0.89 |
| Monocytes/nl | 0.092 | 0.57 | 0.21 | 0.25 | -0.38 | 0.35 |
| Eosinophils/nl | 0.03 | 0.89 | -0.043 | 0.82 | 0.34 | 0.40 |
| Basophils/nl | 0.14 | 0.40 | 0.13 | 0.46 | 0.03 | 0.95 |
| Thrombocytes/nl | -0.28 | 0.08 | -0.41 | 0.31 | -0.16 | 0.39 |
| Neutrophils % | -0.47 | **0.0024** | -0.42 | **0.018** | -0.47 | 0.24 |
| Lymphocytes % | 0.4 | **0.012** | 0.33 | 0.06 | 0.46 | 0.25 |
| Monocytes % | 0.5 | **0.001** | 0.53 | **0.0017** | 0.12 | 0.78 |
| Eosinophils % | 0.15 | 0.37 | 0.06 | 0.76 | 0.77 | 0.026 |
| Basophils % | 0.22 | 0.17 | 0.18 | 0.31 | 0.28 | 0.51 |
|  |  |  |  |  |  |  |
|  | **(SM + VAT)/B** | | | | | |
|  | **All** | | **Male** | | **Female** | |
|  | Correlation | p-value | Correlation | p-value | Correlation | p-value |
| Leukocytes/nl | -0.32 | **0.04** | -0.34 | 0.055 | -0.43 | 0.29 |
| Neutrophils/nl | -0.34 | **0.028** | -0.37 | **0.04** | -0.44 | 0.27 |
| Lymphocytes/nl | 0.26 | 0.10 | 0.25 | 0.16 | -0.03 | 0.95 |
| Monocytes/nl | 0.13 | 0.41 | 0.26 | 0.16 | -0.57 | 0.14 |
| Eosinophils/nl | 0.049 | 0.77 | -0.016 | 0.93 | 0.24 | 0.569 |
| Basophils/nl | 0.1 | 0.55 | 0.09 | 0.62 | -0.1 | 0.81 |
| Thrombocytes/nl | -0.25 | 0.12 | -0.12 | 0.52 | -0.56 | 0.14 |
| Neutrophils % | -0.47 | **0.0024** | -0.42 | **0.018** | -0.51 | 0.19 |
| Lymphocytes % | 0.44 | **0.0041** | 0.4 | **0.024** | 0.52 | 0.19 |
| Monocytes % | 0.43 | **0.006** | 0.43 | **0.013** | 0.11 | 0.79 |
| Eosinophils % | 0.15 | 0.37 | 0.06 | 0.73 | 0.84 | **0.009** |
| Basophils % | 0.17 | 0.28 | 0.13 | 0.49 | 0.31 | 0.45 |

**Table S4:** Univariate and multivariate time-to-progression analysis of clinical parameters and inflammatory markers

| Time to progression analysis |  |  | **Univariate analysis** | | **Multivariate analysis** | |
| --- | --- | --- | --- | --- | --- | --- |
| **Groups** | | **Median time to progression in months (95% CI)** | **HR (95% CI)** | **p-value** | **HR (95% CI)** | **p-value** |
| Sex | Female | 2.93 (0.13 – 13.7) | 1 | **0.038** | 1 | 0.39 |
|  | Male | 12.2 (5.8 – 41.4) | 0.41 (0.18 – 0.95) |  | 0.62 (0.21 – 1.81) |  |
| Age | >70 years | 11.6 (2.7 – .) | 1 | 0.76 | - | - |
|  | ≦ 70 years | 10.2 (2.8 – 32.6) | 1.13 (0.51 – 2.51) |  | - |  |
| p16-status | Negative | 10.17 (2.8 – 13.) | 1 | 0.153 | - | - |
|  | Positive | 16.2 (2.03 - .) | 0.54 (0.23 – 1.3) |  | - |  |
| UICC | 1 | 12.2 (2.6 – .) | 1 | 0.73 | - | - |
|  | 2 | 14.5 (2.3 – .) | 0.93 (0.19 – 4.63) |  | - |  |
|  | 3 | 22.1 (10.13 – .) | 0.96 (0.19 – 4.76) |  | - |  |
|  | 4 | 5.8 (2.43 – 16.2) | 1.63 (0.48 – 5.51) |  | - |  |
| ECOG | 0 | 32.6 (5.8 – .) | 1 | **0.0143** | 1 | **0.043** |
|  | 1 | 10.17 (2.4 – 16.2) | 2.14 (0.8 – 5.45) |  | 1.44 (0.51 – 4.11) |  |
|  | 2 | 2.8 (0.37 – 14.5) | 3.38 (0.93 – 12.15) |  | 1.68 (0.37 – 7.69) |  |
|  | 3-4 | 1.48 (0.1 – 2.3) | 16.48 (3.2 – 84.96) |  | 12.4 (2.07 – 74.44) |  |
| BMI | < 18.5 kg/m^2)^ | 7.5 (0.1 – 20.2) | 1 | 0.56 | - | - |
|  | ≥ 18.5 kg/m^2)^ | 11.6 (3.17 – 32.6) | 0.74 (0.28 – 1.97) |  | - |  |
| Albumin | ≦ 3.4 g/dl | 7.47 (2.33 – 11.6) | 1 | **0.034** | 1 | 0.51 |
|  | > 3.4g/dl | 16.23 (5.83 – .) | 0.44 (0.23 – 0.95) |  | 0.72 (0.27 – 1.92) |  |
| NLR | High (>6.59) | 2.93 (1.2 – 7.47) | 1 | **0.0026** | - | - |
|  | Low (≤6.59) | 32.6 (11.5 - .) | 0.307 (0.14 – 0.67) |  | - |  |
| AISI | High (>1618.4) | 2.7 (0.63 – 3.17) | 1 | **0.006** | - |  |
|  | Low (≤1618.4) | 13.7 (11.5 – 41.4) | 0.312 (0.14 – 0.69) |  | - |  |
| LMR | High (>0.978) | 14.5 (11.53 – .) | 0.237 (0.103 – 0.547) | **0.0012** | 0.173 (0.041 – 0.73) | **0.017** |
|  | Low (≤0.978) | 2.8 (1.2 – 5.8) | 1 |  | 1 |  |
| SIRI | High (>4.61) | 2.8 (0.63 – 5.83) | 1 | **0.001** | - | - |
|  | Low (≤4.61) | 16.3 (11.6 - .) | 0.261 (0.118 – 0.57) |  | - |  |
| CRP | High (>15 mg/L) | 2.9 (2.1 – 11.6) | 1 | **0.0023** | 1 | 0.07 |
|  | Low (≤15 mg/L) | 26.2 (7.67 - .) | 0.287 (0.152 – 0.656) |  | 0.44 (0.18 – 1.07) |  |
| CRP/L | High (>17.49) | 3.17 (2.33 – 13.73) | 1 | 0.055 | - | - |
|  | Low (≤17.49) | 16.23 (5.8 - .) | 0.466 (0.21 – 1.03) |  | - |  |
| dNLR | High (>3.93) | 2.73 (0.37 – 13.73) | 1 | 0.11 | - | - |
|  | Low (≤3.93) | 12.5 (7.47 – 41.4) | 0.511 (0.233 – 1.12) |  | - |  |
| NLPR | High (>0.029) | 2.93 (2.03 – 7.47) | 1 | **0.028** | 1 | 0.4 |
|  | Low (≤0.029) | 13.17 (10.17 - .) | 0.407 (0.186 – 0.888) |  | 0.55 (0.14 – 2.17) |  |
| PLR | High (>318.5) | 2.93 (2.03 – 11.63) | 1 | **0.035** | - | - |
|  | Low (≤318.5) | 14.5 (11.53 - .) | 0.432 (0.198 – 0.944) |  | - |  |
| SII | High (>147.97) | 2.73 (1.2 – 11.63) | 1 | **0.0124** | - | - |
|  | Low (≤147.97) | 14.5 (10.17 - .) | 0.38 (0.176 – 0.805) |  | - |  |
| NER | High (>638.6) | 11.63 (2.7 – 32.6) | 1 | 0.882 | - | - |
|  | Low (≤638.6) | 11.53 (2.8 – 16.23) | 1.06 (0.5 – 2.24) |  | - |  |

Univariate and multivariate time-to-progression analyses of clinical parameters and inflammatory markers. Abbreviations: AISI (Aggregate Index of Systemic Inflammation), BMI (Body mass index), CRP (C-Reactive Protein), CRP/L (C-Reactive Protein/Lymphocyte ratio), dNLR (derived Neutrophil-to-Lymphocyte Ratio), ECOG (Eastern Cooperative Oncology Group), LMR (Lymphocyte-to-Monocyte Ratio), NLPR (Neutrophil-to-Lymphocyte-to-Platelet Ratio), NLR (Neutrophil-to-Lymphocyte Ratio), SIRI (Systemic Inflammatory Response Index), UICC (Union for International Cancer Control).
